# Inborn errors of immunity with atopic phenotypes: A practical guide for allergists

**DOI:** 10.1016/j.waojou.2021.100513

**Published:** 2021-02-22

**Authors:** Riccardo Castagnoli, Vassilios Lougaris, Giuliana Giardino, Stefano Volpi, Lucia Leonardi, Francesco La Torre, Silvia Federici, Stefania Corrente, Bianca Laura Cinicola, Annarosa Soresina, Caterina Cancrini, Gian Luigi Marseglia, Fabio Cardinale, Immunology Task Force of the Italian Society of Pediatric Allergy and Immunology (SIAIP)

**Affiliations:** a Pediatric Clinic, Fondazione IRCCS Policlinico San Matteo, University of Pavia, Pavia, Italy; b Department of Clinical, Surgical, Diagnostic and Pediatric Sciences, University of Pavia, Pavia, Italy; c Department of Molecular Medicine, University of Pavia, Pavia, Italy; d Department of Clinical and Experimental Sciences, Pediatrics Clinic and Institute for Molecular Medicine A. Nocivelli, University of Brescia and ASST-Spedali Civili di Brescia, Brescia, Italy; e Pediatric Section, Department of Translational Medical Sciences, Federico II University, Naples, Italy; f Center for Autoinflammatory Diseases and Immunodeficiency, IRCCS Istituto Giannina Gaslini, Università degli Studi di Genova, Genoa, Italy; g Department of Maternal Infantile and Urological Sciences, Sapienza University of Rome, Rome, Italy; h Department of Pediatrics, Giovanni XXIII Pediatric Hospital, Bari, Italy; i Division of Rheumatology, IRCCS, Ospedale Pediatrico Bambino Gesù, Rome, Italy; j Division of Pediatrics, S. Camillo-Forlanini hospital, Rome Italy; k Department of Experimental Medicine, Sapienza University of Rome, Rome, Italy; l Unit of Pediatric Immunology, Pediatrics Clinic, University of Brescia, ASST-Spedali Civili Brescia, Brescia, Italy; m Department of Systems Medicine, University of Rome Tor Vergata, Rome, Italy; n Academic Department of Pediatrics, Immune and Infectious Diseases Division, Research Unit of Primary Immunodeficiencies, Bambino Gesù; Children's Hospital, IRCCS, Rome, Italy

**Keywords:** Inborn errors of immunity, Primary immunodeficiency, Atopy, Atopic phenotypes, Allergy

## Abstract

Inborn errors of immunity (IEI) are a heterogeneous group of disorders, mainly resulting from mutations in genes associated with immunoregulation and immune host defense. These disorders are characterized by different combinations of recurrent infections, autoimmunity, inflammatory manifestations, lymphoproliferation, and malignancy. Interestingly, it has been increasingly observed that common allergic symptoms also can represent the expression of an underlying immunodeficiency and/or immune dysregulation.

Very high IgE levels, peripheral or organ-specific hypereosinophilia, usually combined with a variety of atopic symptoms, may sometimes be the epiphenomenon of a monogenic disease. Therefore, allergists should be aware that severe and/or therapy-resistant atopic disorders might be the main clinical phenotype of some IEI. This could pave the way to target therapies, leading to better quality of life and improved survival in affected patients.

## Introduction

Inborn errors of immunity (IEI) are a group of mostly monogenic disorders arising from mutations in genes responsible for immune host defense and immunoregulation.^1^^,^^2^ Typical clinical features include recurrent infections, autoimmunity, inflammatory manifestations, lymphoproliferation, and malignancy.^1^ Interestingly, recent evidence suggests that also common allergic symptoms may represent the expression of an underlying immunodeficiency and/or immune dysregulation.^3^ The recognition of IEI in the context of an allergic phenotype is crucial to ensure prompt diagnosis and appropriate treatment aimed to modulate pathophysiological mechanisms and improve clinical symptoms. Indeed, clinical management and expected outcomes are profoundly different from the ones reported for typical allergic conditions. Also, the correct diagnosis could pave the way for targeted therapies.^4^

The article presents a practical approach to diagnose and manage IEI presenting with atopic phenotypes. We will discuss known monogenic disorders leading to IEI with severe atopic phenotypes in humans. Moreover, we will focus on the red flags that need to be considered to suspect these conditions and on differential diagnosis.

## Atopic phenotypes as clinical manifestations of inborn errors of immunity

Relying on the complex interplay between activation and regulation, the immune system has a fundamental role in protecting the host from pathogenic infections while discriminating between self- and non-self antigens.^5^^,^^6^ In this context, allergy, defined as an immune-mediated hypersensitivity reaction, represents an exaggerated immune response against specific non-self antigens, known as allergens. Frequent allergic manifestations include eczema, allergic rhinitis, asthma, and food allergy, and classic testing used to investigate allergic diseases often shows increased serum immunoglobulin (Ig) E and peripheral blood eosinophilia. It is now clear that in some IEI, allergic symptoms may dominate the clinical presentation.^3^^,^^7^^,^^8^ In particular, the allergic triad defined by increased IgE, eosinophilia, and eczema is shared by different IEI that may be misdiagnosed as common allergic diseases.^3^ Also, different and more complex atopic phenotypes have been recently described. Interestingly, the number of newly identified genes associated with IEI has exponentially increased over the last decade. In addition to identifying novel IEI-related genes, it is now clear that distinct clinical phenotypes may be sustained by gain-of-function (GOF) or loss-of-function (LOF) mutations in the same gene. Moreover, different activity degrees of mutant proteins due to hypomorphic and hypermorphic mutations may also cause IEI phenotypic variability.^5^ In this setting, referring to monogenic disorders leading to a predominant allergic inflammation, Milner et al proposed the term “primary atopic disorders”.^9^^,^^10^ The study of these conditions has provided fundamental insights into human immunity and the pathogenesis of allergic diseases.^11^ The main pathways implicated in the development of atopy range from focal defects in immune cells and epithelial barrier function to global changes in metabolism. In particular, they include impaired T-cell receptor (TCR) signaling and cytoskeletal remodeling, TCR restriction, altered cytokine signaling, tolerance failure, cellular metabolic disturbance, mast cell dysregulation, and skin barrier disruption.^12^ A significant goal of investigating heritable single-gene disorders that lead to severe clinical allergic diseases is to unveil fundamental pathways responsible for hypersensitivity that could be targeted to provide novel therapeutic strategies for patients with allergic diseases, syndromic and non-syndromic alike.^9^

## Individual inborn errors of immunity with atopic phenotypes

Focusing on IEI associated with atopic phenotypes, the broad spectrum of clinical and immunological features associated with individual IEI makes it challenging to define a universal classification. According to the predominant clinical and laboratory characteristics, they can be generally classified into six different phenotypes:

[1] Hyper-IgE syndromes (HIES);
[2] Omenn syndrome (OS);
[3] Wiskott-Aldrich syndrome (WAS) and WAS-like conditions;
[4] Immune dysregulation, polyendocrinopathy, enteropathy, X-linked (IPEX) and IPEX-like conditions;
[5] CBM-opathies due to mutations in genes encoding for Caspase recruitment domain (CARD) proteins – B-cell CLL/lymphoma 10 (BCL10) – MALT1 paracaspase (MALT1), altogether known as CBM complexes;
[6] a miscellanea of other IEI presenting with allergic manifestations.

The literature review has been performed employing EMBASE, Pubmed, Scopus, and Web of Science databases, retrieving all publications on IEI with atopic phenotypes. The search strategy was performed using a free-text search (keywords: inborn errors of immunity, primary immunodeficiency, atopy, atopic phenotypes, allergy) and thesaurus descriptors search (MeSH and Emtree), adapted for all the selected databases. We searched all articles published up to August 2020. The inclusion criteria for eligible articles were the following: publication in peer-reviewed journals and the English language. Articles were excluded by title, abstract, or full text for irrelevance to the analyzed topic. Lastly, to identify further studies that met the inclusion criteria, the references of the selected articles were also reviewed.

Patients suffering from IEI with atopic phenotypes usually present with peculiar associated clinical manifestations and laboratory findings that need to be carefully analyzed in order to identify the underlying disease. In addition, it is fundamental to assess the presence or absence of a positive family history for primary immunodeficiencies and/or consanguinity, as well as the presence of pre- and perinatal factors that may have influenced the early development of the immune system, including maternal infection during pregnancy.

**Fig. 1:**
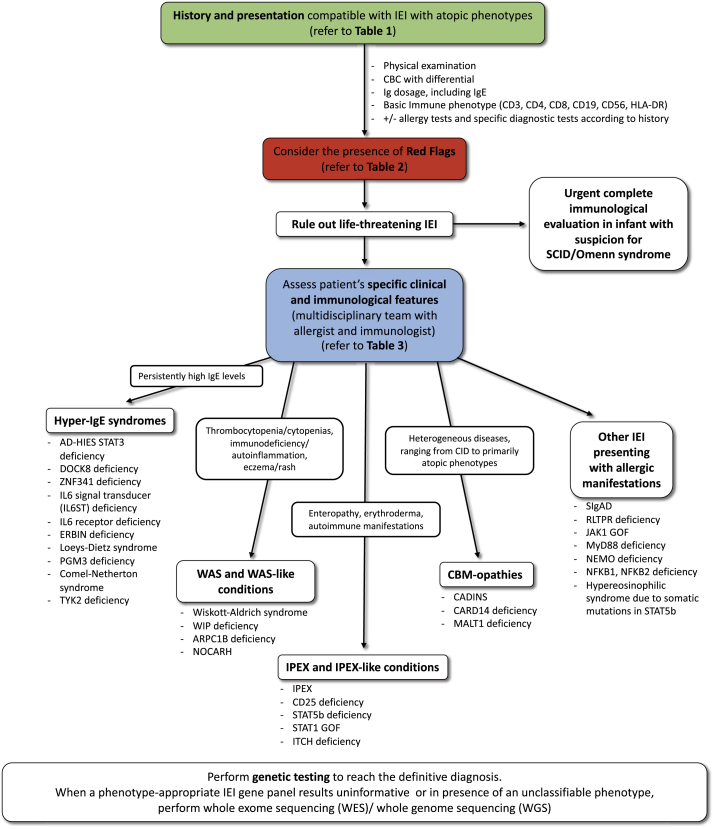
Proposal for a diagnostic algorithm for the identification of IEI with atopic phenotypes

Table 1 and Table 2 summarize the common features of IEI with atopic phenotypes and the red flags that clinicians should consider in the diagnostic work-up, respectively. Table 3 shows an overview of each IEI analyzed in the text, highlighting the distinguishing features from classical allergic disorders. Fig. 1 depicts a proposal for a diagnostic algorithm for the identification of IEI with atopic phenotypes.

**Table 1:** Common features of inborn errors of immunity with atopic phenotypes

Early-onset atopic disease, usually at birth or in the first months of life
Severe atopic disease, usually not responsive to standard therapy (e.g. severe and recalcitrant eczema)
High levels of Th2 biomarkers (e.g. increased total serum IgE, eosinophilia)
Presence of other affected family members (inheritance pattern, including family history for primary immunodeficiencies and/or familial severe atopic diathesis), family history of consanguinity
Associated clinical features^a^
Associated immunological abnormalities^a^
Efficacy of targeted therapies

a See Table 2, Red flags

**Table 2:** Red flags to suspect inborn errors of immunity with atopic phenotypes.

Serum total IgE >2000 kU/L, especially in the first 3 months of life

Neonatal erythroderma
Congenital ichthyosis

AD + Serum total IgE >2000 kU/L + recurrent skin and pulmonary infections ± skeletal abnormalities ± neurodevelopmental delay
Atopic diathesis + recurrent/severe infections (especially due to opportunistic pathogens and Herpesviridae, including CMV, EBV, HHV-6)
AD + autoimmunity ± recurrent infections
Atopic diathesis + lymphopenia
Atopic diathesis + cytopenias (neutropenia/thrombocytopenia/anemia)
AD + diarrhea + endocrinopathy ± failure to thrive
AD + diarrhea + bleeding ± failure to thrive
EGID + severe eosinophilia (>1500 cells/mm3) ± atopic diathesis

AD, atopic dermatitis; CMV, cytomegalovirus; EBV, Epstein-Barr virus; EGID, eosinophilic gastrointestinal disease; HHV-6, Human herpesvirus 6; IgE, immunoglobulin E

**Table 3:** Inborn errors of immunity with atopic phenotypes.

Disease	Genetic defect	Inheritance	Main Features	Distinguishing features from common allergic disorders
**Hyper-IgE syndromes (HIES)**				
AD-HIES STAT3 deficiency (Job syndrome)	*STAT3*	AD LOF	Eczema, skin abscesses, CMC, recurrent pneumonias leading to pneumatocoeles, and skeletal and connective tissue abnormalities	Early-onset eczema; peculiar thickened texture of the facial skin, retroauricular fissures, and severe folliculitis of the axillae and groin; cold abscesses; distinctive facial, and skeletal features, low frequency of allergy

DOCK8 deficiency	*DOCK8*	AR	Severe eczema, severe allergies, immunodeficiency with increased susceptibility to bacterial and viral infections, autoimmunity, and increased risk for malignancies	Severe eczema associated with warts, severe skin and sinopulmonary infections
ZNF341 deficiency	*ZNF341*	AR	Phenocopy of AD-HIES	Same as AD-HIES
IL6 signal transducer (IL6ST) deficiency	*IL6ST*	AR or AD LOF	Largely overlapping with AD-HIES: eczema, recurrent skin and pulmonary infections, craniosynostosis, neurodevelopmental delay	Severe eczema, recurrent cutaneous and pulmonary infections, distinctive skeletal features
IL6 receptor deficiency	*IL6R*	AR	Partially overlapping with AD-HIES: no skeletal abnormalities	Recurrent pyogenic infections, cold abscesses
ERBIN deficiency	*ERBB2IP*	AD LOF	Eczema, eosinophilic esophagitis, skeletal and connective tissue abnormalities like STAT3-HIES	Skeletal and connective tissue abnormalities
Loeys-Dietz syndrome (TGFBR deficiency)	*TGFBR1 TGFBR2*	AD	Marfan-like syndrome, high prevalence of allergic diseases	Skeletal and connective tissue abnormalities
PGM3 deficiency	*PGM3*	AR	Skeletal dysplasia, immunodeficiency and tendency to bone marrow failure, severe atopy, neurodevelopmental delay; some patients display renal, intestinal, and heart defects.	Complex syndromic phenotype associated with atopy
Comel-Netherton syndrome	*SPINK5*	AR	Congenital ichthyosis, bamboo hair, atopic diathesis; increased bacterial infections; enteropathy, failure to thrive	Congenital ichthyosis
TYK2 deficiency	*TYK2*	AR	Susceptibility to intracellular bacteria (mycobacteria, Salmonella) and viruses; dermatitis	Peculiar susceptibility to infections

**Omenn syndrome**				
OS is associated with multiple genetic abnormalities	*RAG1, RAG2, IL2RG, IL7R, LIG4, ADA, DCLRE1C, RMRP, CHD7, ZAP70,* *22q11del* and more	AR, XL	Erythroderma, lymphadenopathy, eosinophilia, and combined immunodeficiency	Erythroderma or neonatal eczematous rash; immunodeficiency
**Wiskott-Aldrich syndrome (WAS) and WAS-like conditions**				
Wiskott-Aldrich syndrome	*WAS*	XL	Thrombocytopenia, recurrent infections, eczema, bloody diarrhea, haematological malignancies, autoimmune manifestations	Eczema associated with thrombocytopenia and recurrent infections
WIP deficiency	*WIPF1*	AR	Thrombocytopenia with or without small platelets, recurrent infections, eczema, bloody diarrhea	WAS-like phenotype
ARPC1B deficiency	*ARPC1B*	AR	Mild thrombocytopenia, recurrent infections, autoimmunity; dermatitis	WAS-like phenotype
NOCARH	*CDC42*	AD	Neonatal-onset cytopenia, autoinflammation, rash, and episodes of hemophagocytic lymphohistiocytosis; wide phenotypic heterogeneity	Autoinflammation, cytopenia, episodes of HLH
**Immunodysregulation, polyendocrinopathy, enteropathy, X-linked (IPEX) and IPEX-like conditions**				
IPEX	*FOXP3*	XL	Autoimmune enteropathy, early onset diabetes, thyroiditis, hemolytic anemia, thrombocytopenia, severe early-onset dermatitis, recurrent severe infections, elevated IgE and IgA	Severe early-onset dermatitis associated with multiorgan autoimmunity
CD25 deficiency	*IL2RA*	AR	IPEX-like syndrome; chronic viral, fungal, and bacterial infections	IPEX-like syndrome
STAT5b deficiency	*STAT5B*	AR or AD LOF	Growth-hormone insensitive dwarfism; dysmorphic features; eczema; prominent autoimmunity Growth-failure; eczema (no immune defects compared to AR STAT5b deficiency)	IPEX-like syndrome, dwarfism, dysmorphic features
STAT1 GOF	*STAT1*	AD GOF	CMC, infections, autoimmunity (thyroiditis, diabetes, cytopenias), enteropathy	CMC, autoimmunity
ITCH deficiency	*ITCH*	AR	Autoimmunity, failure to thrive, developmental delay, dysmorphic facial features	Autoimmunity, dysmorphic facial features
**CBM-opathies**				
CADINS	*CARD11*	AD LOF	Atopic disease, respiratory tract infections and cutaneous viral infections Increased IgE, eosinophilia, Th-2 skewed immune response	Severe atopic disease associated with susceptibility to infections and immune dysregulation
CARD14 deficiency	*CARD14*	AD LOF	Atopic disease, recurrent pyogenic and viral skin infections and respiratory tract infections	See CARD11
MALT1 deficiency	*MALT1*	AR	Recurrent infections of the skin and of the respiratory and gastrointestinal tracts, failure to thrive, periodontal disease and inflammatory gastrointestinal disease	Recurrent infections and inflammatory gastrointestinal disease
**Other IEI presenting with atopic phenotypes**				
Selective IgA deficiency (SIgAD)	Unknown	Unknown	Frequently asymptomatic. Susceptibility to infections, autoimmunity and allergy Serum IgA levels (<0.07 g/L), normal serum IgG and IgM on at least two determinations	Isolated IgA deficiency
RLTPR deficiency	*CARMIL2*	AR	Recurrent infections, EBV lymphoproliferation and other malignancy, atopy	Infections, atopy, malignancies
JAK1 GOF	*JAK1*	AD GOF	Eosinophilia, hepatosplenomegaly, eosinophilic enteritis, poor growth, viral infections	Hypereosinophilic syndrome
MyD88 deficiency	*MYD88*	AR	Bacterial infections (pyogens), high IgE levels	Peculiar susceptibility to pyogenic infections
EDA-ID due to IKBKG (NEMO) deficiency	*IKBKG (NEMO)*	XL	Anhidrotic ectodermal dysplasia; susceptibility to infections (bacteria, mycobacteria, viruses, fungi)	Peculiar phenotype of anhidrotic ectodermal dysplasia
NFKB1 deficiency	*NFKB1*	AD	Recurrent respiratory infections, EBV proliferation, autoimmunity	Susceptibility to infections, autoimmunity (cytopenias, alopecia, thyroiditis)
NFKB2 deficiency	*NFKB2*	AD	Recurrent respiratory infections, autoimmunity	Susceptibility to infections, autoimmunity (alopecia and endocrinopathies)
Hypereosinophilic syndrome due to somatic mutations in STAT5b	*STAT5B* (GOF) – somatic mutations	–	Eosinophilia, atopic dermatitis, urticarial rash, diarrhea	Hypereosinophilic syndrome

AR, autosomal recessive; AD, autosomal dominant; CADINS, CARD11-associated atopy with dominant interference of NF-kB signaling; CMC, chronic mucocutaneous candidiasis; EDA-ID, Anhidrotic Ectodermal Dysplasia with ImmunoDeficiency; GOF, gain of function; HLH, hemophagocytic lymphohistiocytosis; LOF, loss of function; XL, X-linked

### Hyper-IgE syndromes

IgE antibodies play a central role in the pathogenesis of atopic diseases and in host immunity against parasitic infections. Serum IgE levels in non-atopic subjects are usually very low (0–200 IU/mL)^13^^,^^14^ but vary significantly according to age and ethnicity.^15^^,^^16^ Atopic patients have elevated antigen-specific and total serum IgE levels (1000–10,000 IU/mL).^17^ It is now well established that different IEI can manifest with elevated serum IgE as a sign of immune dysregulation.^7^^,^^18^ Classically, and up to recent years, markedly elevated serum IgE levels have been the hallmark of HIES. Moreover, many other IEI, including WAS, IPEX, Omenn syndrome, and atypical DiGeorge syndrome are characterized by an increase in serum IgE (see described below).

**Fig. 2:**
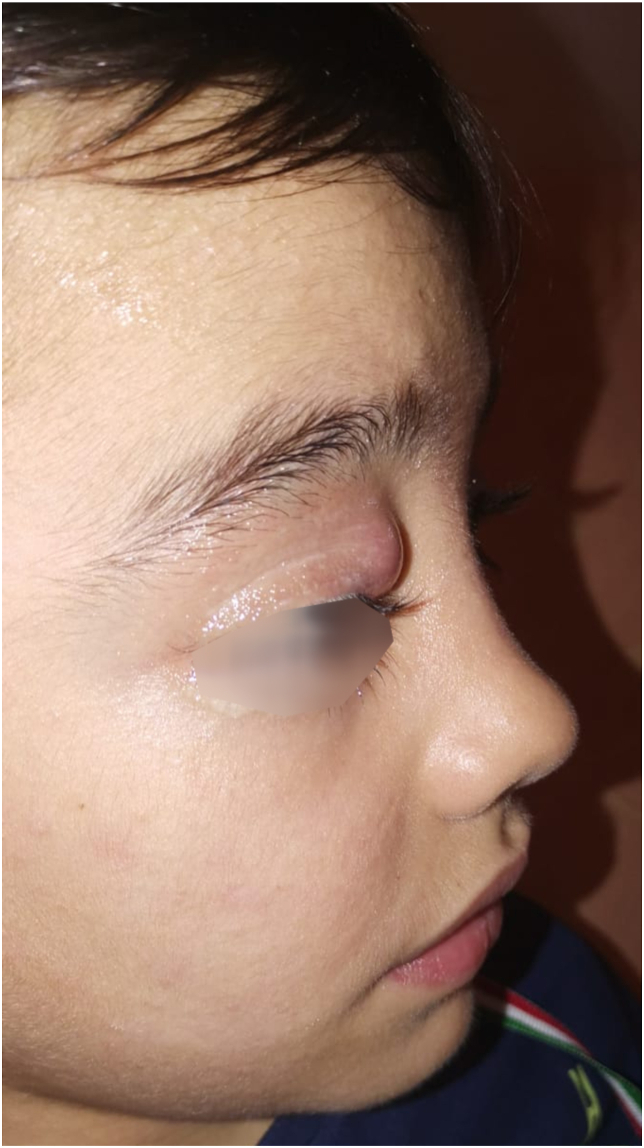
Upper eyelid abscess in a patient with STAT3-HIES

Focusing on HIES, the prototypic syndrome is caused by dominant-negative germline mutations in Signal transducer and activator of transcription 3 (*STAT3*)*,* resulting in an autosomal dominant Hyper-IgE (AD-HIES or STAT3-HIES) syndrome, formerly known as Job syndrome, characterized by eczema, skin abscesses [Fig. 2], recurrent pneumonia leading to pneumatocoeles, and skeletal and connective tissue abnormalities, such as bone fragility, scoliosis, and decidual teeth retention.^14^^,^^19^^,^^20^ Other reported manifestations include an increased incidence of both Hodgkin and non-Hodgkin lymphomas;^21^^,^^22^ vascular abnormalities as aneurysms, dilation, and tortuosity of middle-sized arteries such as coronary and cerebral arteries;^23^ gastrointestinal disease as dysmotility, gastro-esophageal reflux, and eosinophilic esophagitis.^24^ The most typical laboratory finding is an elevated serum IgE level (often higher than 2000 IU/mL). Eosinophilia can be observed at the complete blood count (CBC). Immunoglobulin levels are usually normal, but specific antibody responses to encapsulated bacteria can be impaired.^25^ Lymphocyte phenotyping often reveals diminished memory T and B cells and very low IL-17 producing T cells.^25^ The National Institutes of Health (NIH)-scoring system has been developed and validated to support clinicians in the recognition and diagnosis of STAT3-HIES.^13^ Compared to atopic dermatitis (AD), skin findings in STAT3-HIES are characterized by the peculiar thickened texture of the facial skin, retro auricular fissures, and severe folliculitis of the axillae and groin; these skin manifestations appear very early in life (first month) and may sometimes be already present at birth.^26^ The possible presence of chronic mucocutaneous candidiasis (CMC) in patients with STAT3-HIES is another distinguishing feature from AD.^27^ Also, STAT3-HIES manifests with poor clinical and biological inflammation, predisposing to the development of cold abscesses of the skin and lungs. Paradoxically, despite extremely high total serum IgE levels, specific IgE values and skin prick testing are often negative, and STAT3-HIES patients tend to present with lower lifetime frequency and severity of food allergy than AD patients.^28^^,^^29^ The discordance between total IgE and allergic symptoms is at least partially explained by the essential role of STAT3 signaling in mast cell degranulation.^29^ Prophylactic therapy with anti-staphylococcal and antifungal agents and topical antiseptics are fundamental to reduce the risk of cutaneous and sinopulmonary bacterial infections.^25^ The role of hematopoietic stem cell transplantation (HSCT) in treating STAT3-HIES is still under investigation, with encouraging reports on improvement in immunologic and nonimmunologic features of the underlying disease.^30^

**Fig. 3:**
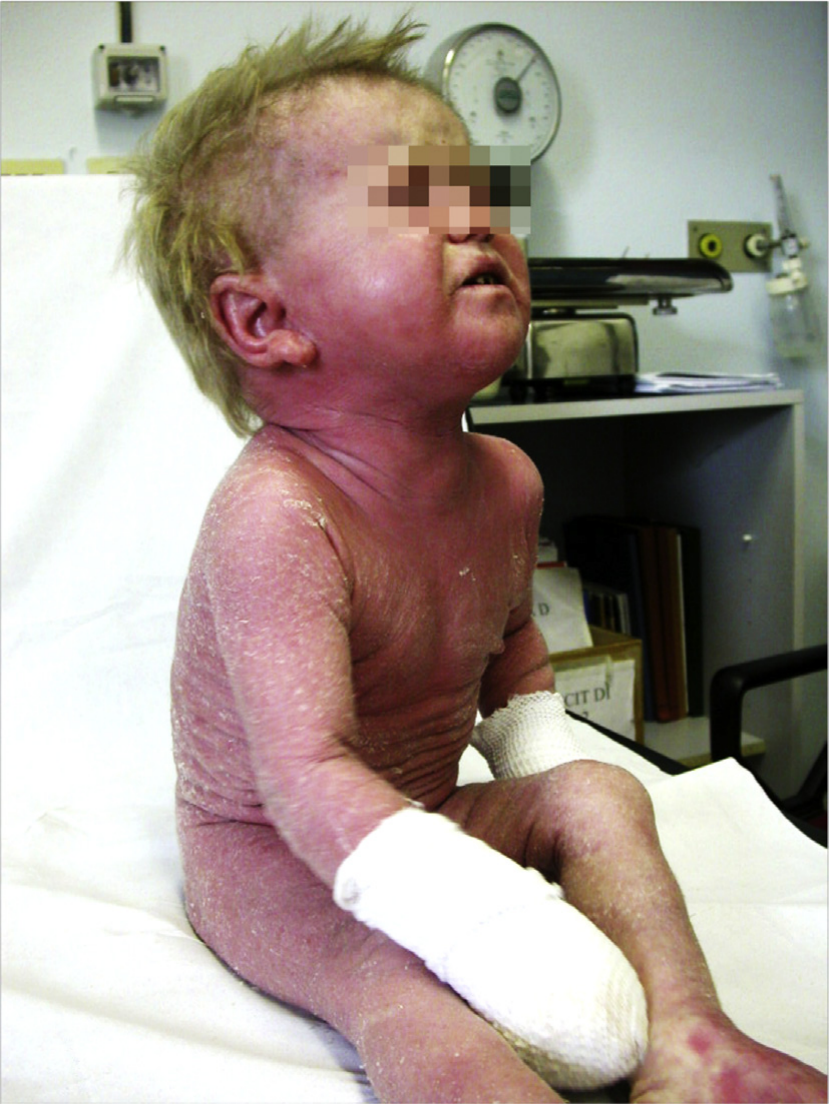
Severe eczema in a patient with DOCK8 deficiency

When severe eczema is associated with recurrent viral infections, a combined immunodeficiency (CID) syndrome should also be considered. Dedicator of Cytokinesis 8 (DOCK8) deficiency is an autosomal recessive CID presenting with severe eczema [Fig. 3], severe allergies, immunodeficiency with increased susceptibility to bacterial, fungal, and viral infections, autoimmunity, neurological manifestations, cerebral vascular malformations and increased risk for malignancies.^31^^,^^32^ Although some clinical features overlap with STAT3-HIES, including severe eczema, skin, and sinopulmonary infections, elevated IgE, and eosinophilia, DOCK8 deficiency mainly differs for (i) susceptibility to cutaneous viral infections such as human papillomavirus (HPV) causing diffuse warts, disseminated molluscum contagiosum, herpes simplex viruses; (ii) a higher frequency of allergic manifestations including atopic dermatitis, food allergies, asthma, and eosinophilic esophagitis; (iii) the risk of malignancies that can be secondary to poor control of viruses such as HPV-associated squamous cell carcinomas or not associated with viral infections as rapidly progressive T-cell lymphoma; (iv) no predisposition to develop pneumatoceles, fractures, scoliosis or to retain teeth.^25^^,^^33^, ^34^, ^35^ At present, HSCT is the only curative option for DOCK8 deficiency and is recommended at the early stages of the disease.^36^ Interestingly, not all disease-related manifestations responded equally well to transplantation: infections and eczema resolved quicker than food allergies.

A novel autosomal recessive (AR) form of HIES was described in 2018; it is due to biallelic mutations in Zinc Finger Protein 341 (*ZNF341*), a transcription factor that regulates the transcription of *STAT3*, thereby also regulating its expression and activity.^37^^,^^38^

Moreover, Schwerd et al reported that severely hypomorphic mutations of the Interleukin 6 Signal Transducer (*IL6ST*) gene are also responsible for a severe AR form of HIES.^39^ Interestingly, the *IL6ST* gene encodes for the gp130 co-receptor of IL-6 family cytokines that include IL-6, IL-11, IL-27, and transduce the signal via STAT3. Recently, heterozygous, dominant-negative mutations in *IL6ST* have been described as the second genetic etiology of autosomal dominant HIES.^40^

Although only a few cases have been described carrying these recently identified mutations, it seems that the clinical phenotypes of the patients with these genetic etiologies of HIES mostly, but not entirely, overlap.^5^ In contrast, patients with autosomal recessive Interleukin 6 Receptor (IL-6R) deficiency,^41^ although presenting with similar clinical features, do not display skeletal phenotypes.

The significant overlap between allergic and connective tissue features has been better-understood thanks to Lyons et al., who, in 2017, reported a family with a loss-of-function (LOF) mutation in *ERBB2IP*, which encodes for the ERBB2-interacting protein (ERBIN).^42^ ERBIN deficiency presents with elevated IgE, recurrent respiratory infections, eosinophilic esophagitis, joint hypermobility, and vascular abnormalities; these patients do not manifest mucosal susceptibility to candida and T- and B-cell memory impairment as observed in STAT3-HIES. Of note, it is now known that ERBIN is fundamental for STAT3-mediated downregulation of Transforming Growth Factor Beta (TGF-β) signaling. Loss of ERBIN induces T-regulatory cell proliferation and Th2 polarization, recapitulating the allergic and connective tissue phenotypes of STAT3-HIES.^42^

The same molecular pathway is involved in the pathogenesis of Loeys-Dietz syndrome due to autosomal dominant mutations in the TGF-β receptor pathway.^43^ Affected individuals present with a Marfan-like syndrome, familial thoracic aortic aneurysms, and high prevalence of allergic manifestations, including eczema, food allergy, asthma, allergic rhinitis, and eosinophilic gastrointestinal disease.

Among HIES, complex and widespread clinical manifestations are reported in patients with Phosphoglucomutase 3 (PMG3) deficiency.^44^ The enzyme PMG3 is involved in multiple glycosylation pathways, and *PMG3* mutations cause an AR disease characterized by severe skeletal dysplasia, severe atopy, and autoimmunity along with immunodeficiency and tendency to bone marrow failure, often associated with neurodevelopmental delay; moreover, some patients display renal, intestinal, and heart defects.

Although previously not considered among primary immunodeficiencies, Comèl-Netherton syndrome is now included in the IUIS classification of IEI.^1^^,^^45^^,^^46^ It is a congenital ichthyosis syndrome caused by AR mutations in the serine protease inhibitor gene Kazal-type 5 (*SPINK5*), which plays a pivotal role in maintaining skin barrier integrity.^47^ Comèl-Netherton syndrome is characterized by an early-onset generalized rash that evolves into severe ichthyosis with typical bamboo hair (trichorrhexis invaginata).^45^ Along with skin disease, these patients present with enteropathy and recurrent bacterial infections.^46^ In particular, Renner et al reported impaired vaccine responses, particularly to polysaccharide vaccines.^46^ Comèl-Netherton syndrome is also classified among the inherited skin disorders sharing pathogenetic pathways with atopic conditions^48^ together with ichthyosis vulgaris caused by null mutations in Filaggrin (*FLG),*^49^ the inflammatory peeling skin syndrome due to mutations in Corneodesmosin (*CDSN),*^50^ the severe skin dermatitis, multiple allergies and metabolic wasting (SAM) syndrome due to bi-allelic mutations in *DSG1,* encoding the desmosomal cadherin desmoglein 1 (DSG1),^51^ or in *DSP*, encoding another desmosomal protein, desmoplakin.^52^ Interestingly, a functional role for DSG1 and its dysregulation in the pathophysiology of eosinophilic esophagitis has been reported.^52^^,^^53^ Moreover, a favorable response to the treatment with ustekinumab, a monoclonal antibody targeting IL-12 and IL-23 as well as downstream IL-17 pathways, has been recently described in patients with *DSP* mutations^54^^,^^55^

Further data are needed to evaluate if these other barrier defects may be associated with immunodeficiency.^12^

Tyrosine kinase 2 (TYK2) deficiency was formerly defined in a patient suffering from an autosomal recessive form of HIES.^56^ Minegishi et al described a 22-year-old Japanese male patient who displayed the characteristic features of HIES associated with susceptibility to various pathogens, including mycobacteria and herpes simplex virus.^56^ More recently, the comprehensive immunological investigation of other TYK2-deficient patients has revealed a wider spectrum of disease, including phenotypes with mycobacterial and viral infections without hyper-IgE syndrome.^57^, ^58^, ^59^, ^60^

According to the 2019 IUIS classification of IEI, also heterozygous dominant-negative mutations in Caspase Recruitment Domain Family Member 11 (*CARD11*) cause a Hyper-IgE syndrome. However, considering the specific molecular pathway involved in the disease, it will be discussed among CMB-opathies (see below).

### Omenn syndrome

Omenn syndrome (OS) was first described in 1965, in infants who presented with generalized erythroderma, lymphadenopathy, eosinophilia, and CID.^61^ Although this condition has been initially associated with mutations in Recombination activating gene 1 and 2 (*RAG1* and *RAG2*),^62^^,^^63^ genetic alterations in other genes have also been reported,^64^, ^65^, ^66^, ^67^, ^68^, ^69^, ^70^, ^71^, ^72^ including the ones responsible for ARTEMIS deficiency, ADA deficiency, Cartilage Hair Hypoplasia, CHARGE syndrome, EXTL3 deficiency and atypical complete DiGeorge syndrome. Moreover, leaky severe combined immunodeficiency (SCID) caused by hypomorphic mutations in the common γ-chain (IL-2 receptor γ), IL-7 receptor α, ZAP70^,^ and DNA ligase 4 may present with an OS phenotype. It is now clear that OS is not an isolated form of CID and is not caused by a single genetic defect.^73^ Instead, it is an exaggerated inflammatory condition that can be caused by different genetic alterations that significantly reduce, but do not abrogate, T cell development, resulting in an oligoclonal expansion of CD4^+^ T cells.

With regards to the atopic manifestations of OS, the disease usually presents at birth with generalized erythroderma, defined as skin inflammation affecting more than 90% of the body surface.^74^ Differential diagnosis of a newborn with erythroderma includes infections, inborn errors of metabolism, ichthyoses and inflammatory skin disorders, drug hypersensitivity reactions, and congenital immunodeficiencies.^26^ Of note, although the initial cutaneous manifestation of OS is most commonly described as erythroderma, it may present with a neonatal eczematous rash.^75^ Early recognition of OS is fundamental to allow for early HSCT that is the only curative treatment for this otherwise fatal disease.^76^ The diagnostic work-up should include an immunological evaluation with immune phenotype analysis and immunoglobulin dosage. Although IgGs are delivered to the infant through the placenta, this is not true for IgA and IgM. Thus, correct evaluation of all Ig isotypes should be performed and should always be confronted with age-matched values. Moreover, it is fundamental to consider the possibility of maternal engraftment that can confound the diagnostic process. Although basic flow cytometry evaluation (CD3, CD4, CD8, CD19, CD56, and HLA-DR expression) may be able to indicate a maternal engraftment by excessive expression of HLA-DR on patients' T cells — indicative in the context of CID suspicion of maternal origin — the variable number of tandem repeat (VNTR) analysis, also referred to as microsatellite analysis and/or *in situ* hybridization, represents the current gold standard to evaluate the maternal engraftment. VNTR probes give strong hybridization signals allowing for earlier detection of chimerism as well as detection of small numbers of cells.^77^

Among SCID, atopy and eosinophilia are frequently reported in Adenosine deaminase (ADA)-SCID. Allergic rhinitis and asthma, atopic dermatitis, urticaria and food allergy are the most common atopic manifestations identified in this population.^78^^,^^79^

### Wiskott-Aldrich syndrome (WAS) and WAS-like conditions

Wiskott-Aldrich syndrome (WAS) is an X-linked IEI typically presenting with the triad of immunodeficiency, eczema, and thrombocytopenia with small platelets (mean platelet volume, MPV <6 fL). Recurrent and/or chronic infections, autoimmune manifestations, and increased susceptibility to malignancies, especially EBV-associated lymphoma, represent the main features of the syndrome.^80^ With an estimated incidence of 1 in 100 000 live male births, WAS is caused by mutations in the *WAS* gene, encoding the WAS protein (WASP), mainly involved in signal transduction and cytoskeleton remodeling. WASP plays a pivotal role in the immunological synapse formation and in the migration of myeloid and lymphoid cells in response to chemotactic signals.^81^ A broad spectrum of clinical phenotypes has been described in patients with *WAS* mutations. Of note, it is now known that hypomorphic mutations of *WAS* cause isolated X-linked thrombocytopenia (XLT),^82^ which may even be intermittent^83^; moreover, gain-of-function (GOF) mutations in the GTPase-binding domain of WASp are responsible for isolated X-linked congenital neutropenia.^84^

**Fig. 4:**
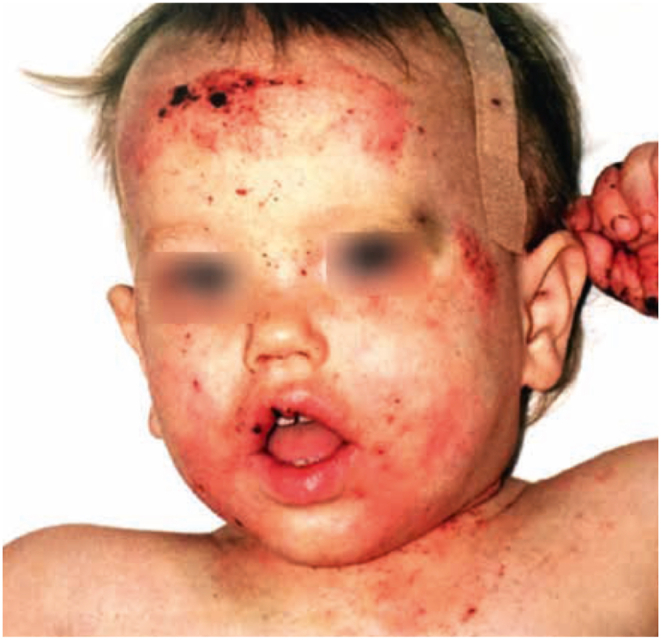
Severe eczema, petechiae and purpura in a patient with Wiskott-Aldrich syndrome

Typically, patients suffering from WAS present early in life with severe eczema, bloody diarrhea, and recurrent infections.^80^ Bacterial respiratory infections are common; patients are also at risk for chronic viral infections, particularly caused by herpesviruses, papillomavirus, and molluscum contagiosum. Autoimmunity usually manifests with hemolytic anemia, inflammatory bowel disease, arthritis, and IgA nephropathy. Increased risk of EBV-driven lymphoproliferative disease, lymphoma, and leukemia is reported. Progressive lymphopenia with impaired T-cell proliferation, altered NK-cytolytic function, decreased levels of IgM with increased IgA and IgE, impaired production of antibodies (especially to polysaccharide antigens), and reduced number of switched memory B cells represent the main immunological features.^80^ As for the atopic phenotype, eczema has been reported in 81% of patients with WAS [Fig. 4].^85^ Eczema may resemble classical AD but is usually more severe and widespread, is associated with petechiae and purpura due to the hemorrhagic diathesis, and typically present during the first year of life. Antimicrobial prophylaxis and immunoglobulin replacement, if required, represent the mainstays of supportive therapy, while HSCT and gene therapy represent the curative treatment.^86^^,^^87^

A WAS-like phenotype has been reported in patients with WIP deficiency and ARPC1B deficiency, in both cases associated with congenital thrombocytopenia.

WASP-interacting protein (WIP) is fundamental for WASP molecular stabilization^88^ and is part of the DOCK8-WIP-WASP complex that links the T-cell receptor (TCR) to the actin cytoskeleton.^89^ Mutations of the *WIPF1* gene, causing WIP deficiency, are responsible for an IEI resembling WAS,^90^ with eczema being reported in most patients.^91^

**Fig. 5:**
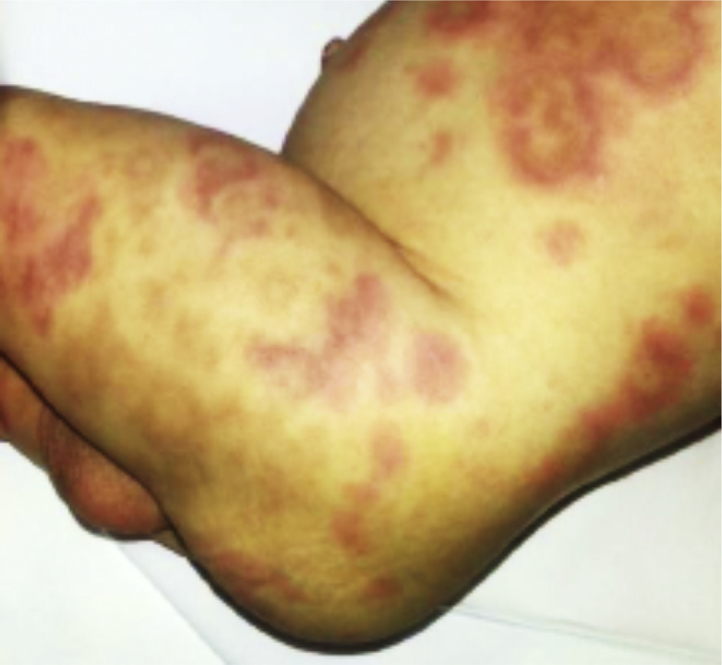
Cutaneous rash associated with macrophage activation syndrome (MAS) in a patient with ARPC1B deficiency (adapted from Brigida et al.^96^

ARPC1B deficiency is an AR form of CID associated with immune dysregulation and platelet abnormalities.^92^, ^93^, ^94^ The Actin-Related Protein Complex 1B (ARPC1B) is required for the assembly and maintenance of the ARP2/3 complex that plays a pivotal role in actin branching. ARPC1B-deficient patients present with clinical and laboratory features suggestive of WAS, including dermatitis, thrombocytopenia with bloody diarrhea, vasculitis, recurrent infections, autoimmune and atopic diathesis^95^^,^^96^; in addition, episodes of macrophage activation syndrome have been reported in these patients [Fig. 5].^94^^,^^96^

A disease characterized by neonatal-onset cytopenia, autoinflammation, rash, and episodes of hemophagocytic lymphohistiocytosis (NOCARH) has been recently described in 4 unrelated patients carrying the same de novo heterozygous missense mutation in Cell division cycle 42 (*CDC42*) at p.Arg186Cys.^97^ CDC42 is a member of the Ras-homologous (Rho) GTPase family, functioning as a signaling node controlling a number of cellular processes, including proliferation, migration and adhesion.^98^^,^^99^ Interestingly, NOCARH differs considerably from the conditions previously associated with CDC42 mutations that showed a heterogeneous collection of neurodevelopmental phenotypes, including Takenouchi-Kosaki syndrome.^100^^,^^101^ More recently, additional reports further expanded the clinical spectrum of human diseases caused by inherited *CDC42* mutations.^102^, ^103^, ^104^, ^105^, ^106^, ^107^, ^108^ Of note, He et al and Bekhouche et al reported on 2 patients with the p.Arg186Cys mutation, dysmorphism, and NOCARH along with elevation in total serum IgE.^107^^,^^108^

### Immune dysregulation, polyendocrinopathy, enteropathy, X-linked (IPEX) and IPEX-like conditions

Immune dysregulation, polyendocrinopathy, enteropathy, X-linked (IPEX) is a X-linked recessive IEI that manifests in infancy with enteropathy, eczema, and severe autoimmune manifestations, including cytopenias, type 1 diabetes mellitus, autoimmune hepatitis, nephropathy, and myopathy.^109^^,^^110^ IPEX is caused by mutations in the *FOXP3* gene, encoding for the Forkhead box protein 3, which is fundamental for regulatory T (Treg) cell function and immune tolerance.^110^ Immune abnormalities include lack of CD4^+^ CD25^+^ FOXP3+ Treg cells, eosinophilia, elevated serum IgE, and increased levels of autoantibodies.^111^^,^^112^ The most frequent atopic feature is severe eczematous dermatitis. However, other less common cutaneous manifestations may be present, including erythroderma, psoriasiform dermatitis, urticaria, pemphigoid nodularis, and alopecia universalis.^113^ Moreover, IPEX patients present with an increased incidence of food allergies.^114^ IPEX is usually fatal if not adequately treated. Medical management of IPEX with immunosuppressive agents such as tacrolimus and rapamycin may alleviate symptoms of the disease, but also expose patients to an increased risk of infections.^115^ Reports on HSCT have shown encouraging results, but it is fundamental to transplant before organ damage develops.^115^

As reported for WAS and WAS-like conditions, several IPEX-like syndromes have been described in the last years,^116^ including CD25 deficiency, STAT5b deficiency, and Itchy E3 Ubiquitin Protein Ligase (ITCH) deficiency [Table 3]. Moreover, gain-of-function mutations (GOF) in STAT1, generally associated with mucocutaneous candidiasis, may manifest as an IPEX-like phenotype. Even though the presentation of these diseases shares many features with IPEX, clinical manifestations specific of each disorder may support the differential diagnosis.^116^ For instance, CD25 deficiency also manifests with chronic viral, fungal, and bacterial infections,^117^ while STAT5b deficiency is also characterized by growth-hormone insensitive dwarfism.^118^ Regarding the atopic phenotypes, allergic dysregulation with eczema and food allergy have been variably reported in all these conditions, often associated with elevated IgE levels and evidence of overt Th2 skewing.^116^

### CBM-opathies

Caspase recruitment domain (CARD) proteins – B-cell CLL/lymphoma 10 (BCL10) – MALT1 paracaspase (MALT1), altogether known as CBM complexes, play a key role as signal transducers, favoring inflammatory and immune responses associated to both cell surface and intracellular receptors.^119^^,^^120^

Diseases due to mutations in genes that are part of this complex (termed CBM-opathies) are extremely heterogeneous and present with a wide variety of clinical manifestations, ranging from CID to primarily atopic phenotypes.^119^^,^^120^ In particular, germline CBM-opathies typically manifest with early-onset, severe atopic diseases include those carrying germline mutations affecting *CARD11*, *CARD14*, and *MALT1*.^119^^,^^120^

While complete LOF mutations in *CARD11* cause profound CID^121^^,^^122^ and heterozygous GOF mutations cause an immunodeficiency associated to B-cell lymphoproliferative disease and referred to as B-cell expansion with NF-kB and T-cell anergy (BENTA),^123^, ^124^, ^125^, ^126^, ^127^ heterozygous dominant-negative mutations are responsible for a distinctive clinical entity called CARD11-associated atopy with dominant interference of NF-kB signaling (CADINS).^128^, ^129^, ^130^, ^131^ The most typical clinical manifestations reported in patients with CADINS include atopic disease, respiratory tract infections, and cutaneous viral infections.^129^ Nearly 90% of patients with CADINS present atopic diseases, with AD and asthma being the most frequent, followed by allergic rhinoconjunctivitis, food allergy, and eosinophilic esophagitis.^129^ Partial clinical overlap with previously described IEI has been reported in some patients with CADINS: atopy and viral infections (DOCK8 deficiency), skeletal abnormalities as retained teeth (STAT3-HIES), failure to thrive, diarrhea, and severe atopic dermatitis (IPEX).^120^ Besides, similar to these conditions, increased IgE, eosinophilia, and Th-2 skewed immune response are frequently observed in CADINS. Immunological phenotype is characterized by normal absolute T- and NK-cell numbers with normal/low B-cell numbers; T-cell proliferation is impaired and hypogammaglobulinemia with altered specific antibody response has been reported.^129^ Antimicrobial prophylaxis and intravenous immunoglobulin can be considered depending on the patient's immune profile and infectious history.^129^ Therapies under investigation include biologics targeting allergic immune dysregulation, such as dupilumab (anti-IL4Rα) or mepolizumab (anti-IL-5)^4^ and glutamine that showed promising *in vitro* results in partially restoring T-cell proliferation.^129^

Recently, Peled et al reported that heterozygous dominant-negative LOF mutations in *CARD14* cause severe atopic dermatitis.^132^ Of note, GOF CARD14 mutations were previously linked to psoriasis and pityriasis rubra pilaris.^133^^,^^134^ Patients with LOF mutations generally manifest with severe atopic dermatitis along with other atopic features, including markedly increased serum IgE levels, asthma, allergic rhinitis, and food allergies. Susceptibility to recurrent pyogenic and viral skin infections and respiratory tract infections is also commonly described in these patients.

Finally, patients carrying biallelic LOF mutations in *MALT1* may present with atopic diseases, mainly dermatitis^135^, ^136^, ^137^, ^138^; however, most frequent clinical manifestations include recurrent infections of the skin and of the respiratory and gastrointestinal tracts, failure to thrive, periodontal disease and inflammatory gastrointestinal disease.

### Other IEI presenting with allergic manifestations

Selective IgA deficiency (SIgAD) has a prevalence in Europe of nearly 1 in 600.^139^ However, the genetic causes underpinning SIgAD are known for a limited number of cases and a clinical/immunologic work-up followed by targeted gene mutation analysis has been proposed for an approach to IgA deficient patients.^140^ Although it is often asymptomatic, SIgA may present with recurrent respiratory infections and autoimmune diseases; moreover, allergic diseases may be the first and/or only clinical manifestation of this condition.^139^

Mutations in *CARMIL2* (Capping Protein Regulator And Myosin 1 Linker 2), also known as *RLTPR* (RGD, leucine-rich repeat, tropomodulin and proline-rich-containing protein) affect the CD28-responsive pathway in T cells and the BCR-responsive pathway in B cells and have been reported in patients with cutaneous and pulmonary allergy, as well as a variety of bacterial and fungal infectious diseases, including invasive tuberculosis and mucocutaneous candidiasis.^141^

Janus kinase 1 *(JAK1)* GOF is responsible for severe atopic dermatitis and hypereosinophilic syndrome characterized by severe eosinophilia with eosinophilic infiltration of the liver and gastrointestinal tract, massive hepatosplenomegaly , autoimmune thyroid disease, and failure to thrive.^142^

Myeloid differentiation primary response protein 88 (MYD88) deficiency is responsible for a Mendelian predisposition to bacterial infections caused principally by pyogenic bacteria. MYD88 is a cytosolic protein recruited by IL-1 receptors (IL-1Rs) and toll-like receptors (TLRs) to trigger the activation of NF-kB pathway and inflammatory cytokine gene transcription. High IgE levels have been reported in patients with MYD88 deficiency, but their correlation with allergic manifestations need to be clearly defined.^143^^,^^144^

NF-kB is a ubiquitous transcription factor member of the Rel proto-oncogene family and regulates the expression of several genes involved in inflammatory and immune responses.^145^ Mutations in genes that affect nuclear factor kB (NF-kB)– dependent signaling are associated with a number of immunodeficiencies including anhidrotic ectodermodysplasia with immunodeficiency (EDA-ID, also known as NEMO deficiency), NFKB1 deficiency and NFKB2 deficiency, in addition to the already described CADINS.^145^, ^146^, ^147^ EDA-ID is characterized by hypotrichosis, hypodontia, hypohidrosis and typical facial features (protruding forehead, characteristic periorbital hyperpigmentation) which are usually associated with immunologic defects such as susceptibility to opportunistic infections, hypogammaglobulinemia, and impaired NK-cell activity.^148^ Heterozygous *NFKB1* gene mutations cause common variable immunodeficiency (CVID)^149^^,^^150^ while *NFKB2* gene defects have been shown to be associated with B cell dysregulation in patients with common variable immunodeficiency (CVID) or combined immunodeficiency (CID)^151^ Among phenocopies of IEI, somatic, GOF STAT5b mutation in a hematopoietic progenitor has been recently reported in 2 patients with a novel syndrome of nonclonal eosinophilia, atopic dermatitis, urticarial rash, and diarrhea.^152^

## Conclusions

Human IEI represent an expanding universe.^5^^,^^153^ In the last 10 years, fundamental insights into the immunopathogenesis of allergic diseases derived from the studies on allergic phenotypes caused by discrete monogenic mutations. Improvement in genetic testing has led to more specific diagnosis and delineation of immune dysregulation syndromes characterized by the hyper IgE phenotype of eczema, recurrent infections, elevated serum IgE and/or hypereosinophilia.

IEI could be misrecognized because of the predominant clinical features of atopy. Without considering an underlying IEI, some individuals will remain undiagnosed, with a high risk of morbidity and mortality. An underlying IEI should be considered, especially in severe cases of atopic diseases with concurrent signs of autoimmunity and recurrent infections, unusual clinical course and lack of response to classical treatment strategies. Common features of IEI with atopic phenotypes and red flags to suspect IEI in the context of atopy should always be carefully considered for every patient. Once suspected, a comprehensive immunological evaluation is required, and genetic testing is essential to identify the specific genetic abnormality.

Integration of knowledge between allergists and immunologists is necessary to make a timely and correct diagnosis of IEI, predict the clinical course, and determine the indication for HSCT and targeted therapies.

## Funding

This work was fully supported by the Italian Society of Pediatric Allergy and Immunology (SIAIP).

## Consent for publication

All the authors give the consent for publication in the journal.

## Ethics approval

Ethics approval was not required for this literature review. Informed consent was obtained from patients to publish the pictures reported in the four figures.

## Author contributions

RC and FC conceived the review and the research method of bibliographic sources. All the authors performed the research, the analysis and the selection of the sources. RC wrote the first draft of the manuscript. All the authors critically revised the manuscript. GLM and FC supervised the project. All the authors accepted the final version of the manuscript.

## Availability of data and materials

All the cited sources are available and reported in the reference list.

## Declaration of competing interest

The authors report no competing interests. The authors have no conflict of interest to disclose with respect to this study.
